# How many human genes can be defined as housekeeping with current expression data?

**DOI:** 10.1186/1471-2164-9-172

**Published:** 2008-04-16

**Authors:** Jiang Zhu, Fuhong He, Shuhui Song, Jing Wang, Jun Yu

**Affiliations:** 1 Key Laboratory of Genome Sciences and Information, Beijing Institute of Genomics, Chinese Academy of Sciences, Beijing, China; 2 Graduate University of Chinese Academy of Sciences, Beijing, China

## Abstract

**Background:**

Housekeeping (HK) genes are ubiquitously expressed in all tissue/cell types and constitute a basal transcriptome for the maintenance of basic cellular functions. Partitioning transcriptomes into HK and tissue-specific (TS) genes relatively is fundamental for studying gene expression and cellular differentiation. Although many studies have aimed at large-scale and thorough categorization of human HK genes, a meaningful consensus has yet to be reached.

**Results:**

We collected two latest gene expression datasets (both EST and microarray data) from public databases and analyzed the gene expression profiles in 18 human tissues that have been well-documented by both two data types. Benchmarked by a manually-curated HK gene collection (HK408), we demonstrated that present data from EST sampling was far from saturated, and the inadequacy has limited the gene detectability and our understanding of TS expressions. Due to a likely over-stringent threshold, microarray data showed higher false negative rate compared with EST data, leading to a significant underestimation of HK genes. Based on EST data, we found that 40.0% of the currently annotated human genes were universally expressed in at least 16 of 18 tissues, as compared to only 5.1% specifically expressed in a single tissue. Our current EST-based estimate on human HK genes ranged from 3,140 to 6,909 in number, a ten-fold increase in comparison with previous microarray-based estimates.

**Conclusion:**

We concluded that a significant fraction of human genes, at least in the currently annotated data depositories, was broadly expressed. Our understanding of tissue-specific expression was still preliminary and required much more large-scale and high-quality transcriptomic data in future studies. The new HK gene list categorized in this study will be useful for genome-wide analyses on structural and functional features of HK genes.

## Background

Human transcriptomes are complicated in three dimensions: diversified to perform tissue/cell-specific functions, undergone temporal regulations during cell cycle and development, and influenced by other physiological and pathological conditions. A collection of genes are expressed in all tissues/cells to maintain basic cellular functions, traditionally known as housekeeping (HK) genes, whereas others are specialized to perform unique functions in differentiated tissues/cells, known as tissue-specific (TS) genes. To characterize cell-specific human transcriptomes, it is important to define this collection of HK genes shared by all human transcriptomes. HK genes were previously considered to express at a constant level across different biological contexts and thus entitled as "control genes" that can be used to standardize quantitative expression studies. However, it has been proven later that the expression of HK genes is still under stringent regulation albeit ubiquitously expressed; their expression levels may vary significantly across different cell types [1-3]. Another related concept refers to "essential genes", the disturbances of which often lead to lethal phenotypes. A recent study has demonstrated that about 500 genes are essential to sustain bacterial life [4]. However, ubiquitous expression does not necessarily mean essentiality and vice versa. In this study, we focused on the primary definition of HK genes — a set of genes universally expressed in diversified tissue/cell types to maintain a basal transcriptome [5].

Previous studies have aimed at large-scale categorization of human HK genes, largely based on microarray technology. There have been three lists of HK genes widely cited in the literature. Warrington et al. [6] and Hsiao et al. [7] pioneered the effort, and obtained 533 and 451 HK genes after sampling 11 and 19 tissues, respectively, by using Affymetrix HuGeneFL chip. Eisenberg et al. [8] later extended the number of HK genes to 575 based on 47 tissue samples, using data from a more advanced Affymetrix U95A platform [9]. Depending on these HK gene lists, many following-up studies have demonstrated distinct natures of HK genes in comparison with TS genes, including gene structure [8,10], nucleotide composition [11], rate of evolution [12,13], protein domain [14], and other genomic characteristics [15-18]. While comparative analyses between HK and TS genes have produced many meaningful results, a consensus on the identity and number of HK genes has been long expected. Although all three microarray-defined HK gene lists arrived at an estimate of about 500 in the number of human HK genes, the overlaps among them were very low.

In this study, we used the latest microarray and EST data from the public databases to re-categorize HK and TS genes. A manually-curated benchmark of HK genes (HK408) was created as control to compare the two different data types. We demonstrated that present EST data was far from saturated and many tissues were still poorly sampled. The inadequacy of EST sampling limited our ability to identify genes and to understand TS expression. The microarray data, due to a likely over-stringent threshold, showed higher false negative rate in comparison with the EST data, leading to a significant underestimation of HK genes. Based on EST data we catalogued a new set of human HK genes, ranging from 3,140 to 6,909 in number, nearly a ten-fold increase as compared to the previous results based on microarray data. We believe that this new dataset will be useful for genome-wide analyses on structural and functional features of HK genes.

## Results

### The limitation of the previous HK gene lists

Three lists of microarray-defined HK genes have been widely cited in the literature. After updating the annotation of these datasets, there were 501, 425, and 567 HK genes in the lists put together by Warrington [6], Hsiao [7] and Eisenberg [8], respectively. Although all of them arrived at an estimate of approximately 500 human HK genes, the shared HK genes were found significantly low — only 155 genes were found in all three datasets despite the fact that two of them shared 340 genes due to the utilization of an identical technical platform (Additional file 1, Figure S1). The unique part of individual dataset ranged from 20% to 60%, implying both high false positive (FP) and false negative (FN) rates in these lists. Moreover, these studies were based on the old microarray platforms with only approximately 7,000 genes represented on the chip, less than half of the present annotations. To update these limited results and avoid systematic bias introduced by a single technique, in this study we analyzed both the latest EST and microarray data to reassess human HK genes.

### Gene expression in microarray and EST data

**Figure 1:**
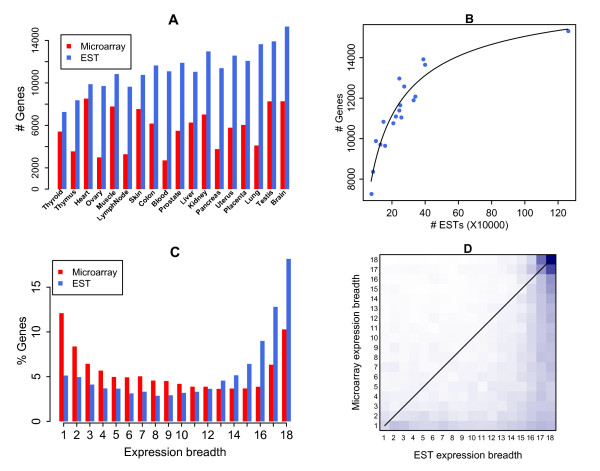
**Gene expression in 18 tissues**. Numbers of genes detected in each tissue are compared between microarray and EST data (A). Tissues are ranked from the poorly-sampled (left) to the highly-sampled (right) according to the EST data. The numbers of detected genes are plotted against the numbers of sampled ESTs for the 18 tissues (B). The sampling growth curve is fitted by Hill function *f*(*x*) = *ax*^*b*^/(*c+x*^*b*^) with *a *= 17622.8, *b *= 0.8, *c *= 6259.7. The curve indicates that current transcriptome sampling is far from saturated. Percentage of genes is plotted against the number of tissues where they express to give the expression breadth distribution (C). Expression breadth in microarray data is compared against that in EST data, with color from white to blue indicating the number of incidence from low to high (D). The correlation of expression breadths between the two types of data is not significant (*r *= 0.42); 71% of the genes are detected in less number of tissues by microarray data than by EST data.

We compiled nearly 8 million human ESTs from 4,026 RNA (or tissue and organ) samples and a recent microarray dataset from Gene Expression Atlas II [19], where 79 RNA (or tissue and organ) samples were hybridized against Affymetrix U133A (coupled with GNF1H) chip. We analyzed the expression of 18,225 RefSeq loci (NCBI, June 18, 2007 update), where 13,986 were represented on the chip. For comparison, we chose 18 well-studied human tissues covered by both data types (Figure 1A); these tissues covered seven major human anatomic systems and should represent a broad spectrum of differentiated tissues/cells in the human body (Additional file 1, Figure S2). In EST data, we defined a RefSeq locus as expressed in a given tissue when at least one reliable EST (singletons) or an EST cluster (contigs) was detected from that tissue, and in microarray data, the expression was defined by the fluorescent intensity when exceeding a cutoff value of 200, as recommended by the authors who carried out the experiments [19] (See methods for details). At the end, we validated the robustness of the conclusions from 18 tissues by extending the analyses to 51 tissues that currently have EST data (Additional file 1, Table S1).

In EST data, we observed that gene detectability in each tissue was proportional to the sampling depth (Figure 1A and 1B). According to the relationship between the number of detected genes and the number of sampled ESTs, the sampling was far from saturated for almost all of 18 tissues (Figure 1B). Many tissues were still very poorly sampled, limiting the gene detectability of current EST data. In microarray data, the number of detected genes was lower than that in EST data, even when ESTs have not been sampled deeply enough (Figure 1A). In our 18-tissue collection, 17,288 of 18,225 total genes (94.9%) were found to be expressed in at least one tissue by EST data, in contrast to 11,730 of 13,986 represented genes (83.9%) by microarray data.

We defined expression breadth as the number of unique tissues where a gene was expressed, which ranged from 1 (TS) to 18 (HK) with decreased tissue-specificity. We observed that the distribution of expression breadth showed two modes representing TS and HK genes respectively in both data types (Figure 1C). The degree of tissue specificity varied gradually and no clear-cut boundaries of both TS and HK genes were observed. However, the expression breadth distributions from the two data types showed opposite trend. In microarray data, majority of genes exhibited tissue-specific expression whereas only a small fraction showed universal expression. 1,418 (12.1%) and 1,206 (10.3%) genes were detected in only one and all 18 tissues, respectively, consistent with previous microarray-based results [8,11]. In EST data, a large fraction of genes was found broadly expressed whereas tissue-specific expression was less notable. 885 (5.1%) genes were detected in only one tissue and 3,140 (18.2%) in all 18 tissues. This was in agreement with a recent microarray experiment on 14 mouse tissues [18].

We compared the expression breadths of 11,495 genes detected in at least one of 18 tissues by both microarray and EST data. The correlation was not significant (Figure 1D, *r *= 0.42); 71% of the genes were detected in less number of tissues by microarray data than by EST data. The above observations implied that microarray data on average detected less number of genes compared with EST data, thus underestimated the expression breadth, making the expression breadth distribution in microarray data skewed toward TS genes.

### Benchmarking housekeeping genes

As universal expression is difficult to testify experimentally, a theoretical definition of HK genes based on annotated universal function is rather desirable. In order to build up a control gene set for a comparative analysis of microarray and EST data, we manually curated 408 genes — a comparable number as previous experimentally-defined HK genes — from large protein complexes or cellular processes that play unquestionable housekeeping roles according to Reactome [20] and KEGG [21] pathway annotations. This included general transcription factors [22,23] and major components of capping and polyadenylation machinery [24-26], spliceosome [27-29], nuclear RNA export complex [30-32], translation machinery [33], cytosolic ribosome [34], and ubiquitin-proteasome proteolytic pathway [35] (Table 1 and Additional file 2). We referred this list as HK408 and used it as a benchmark for evaluating the degree of imperfection in microarray and EST data.

**Table 1:** Functional classification of HK408 genes

**Function**	**Pathway/Complex **^a^	**# Genes**	**MA18 **^b^	**MA16**	**EST18**	**EST16**
Transcription	Transcription pre-initiation complex	40	3	13	12	28
	Basal transcription elongation factor	17	4	7	5	15
	Capping, splicing and polyadenylation	99	42	55	72	96
Transport	Nuclear pore complex	29	2	4	10	26
Translation	Basal translation factor	37	20	28	30	37
	tRNA synthetase	20	8	13	19	20
	Cytosolic ribosome	82	79	79	78	81
Proteolysis	Ubiquitin mediated proteolysis	45	8	12	22	40
	Proteasome	43	17	26	31	41

**Total**		**408**	**182**	**235**	**278**	**379**

^a ^408 genes with highly proposed housekeeping functions are manually curated from Reactome [20] and KEGG [21].

^b ^The column MA18 and MA16 represent the number of these HK genes detected in all 18 tissues and at least 16 tissues by microarray data, respectively; EST18 and EST16 represent that for EST data.

**Figure 2:**
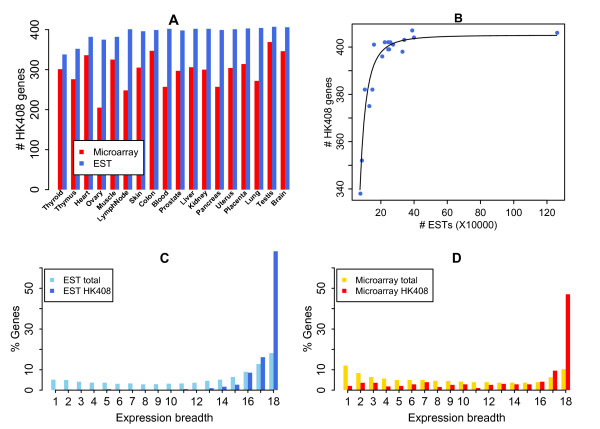
**HK408 gene expression in 18 tissues**. Numbers of HK408 genes detected in each tissue are compared between microarray and EST data (A). Tissues are ranked from the poorly-sampled (left) to the highly-sampled (right) according to the EST data. The numbers of detected HK408 genes are plotted against the numbers of sampled ESTs for the 18 tissues (B). The sampling growth curve is fitted by Hill function *f*(*x*) =* ax*^*b*^/(*c+x*^*b*^) with *a *= 405.0, *b *= 2.4, *c *= 7.0e+10. Five tissues — muscle, ovary, heart, thymus and thyroid — are poorly sampled, primarily accounting for the absence of HK408 genes. The expression breadth of HK408 is predominantly enriched at the value 18 in the EST data (C) whereas a messy tail is observed across all breadth groups in microarray data, indicating a noisy nature and high FP rate (D).

In theory, all HK408 genes should be detected in all 18 tissues if the libraries were sampled adequately. We observed that almost all HK408 genes were detected in EST data of each tissue with only five exceptions (muscle, ovary, heart, thymus and thyroid; Figure 2A), and the poor detection rate in these tissues was primarily due to poor sampling depth (Figure 2B). In contrast, microarray data showed lower detectability compared with EST data (Figure 2A). Comparing each category of HK408 genes, we found that transcription pre-initiation complex (PIC) failed mostly to be universally detected in both microarray and EST data. At best, only 28 of 40 PIC genes were detected in at least 16 tissues in EST data (Table 1). This is believed to reflect the fact that transcription factors are on average expressed at a lower level as compared to other protein complexes, such as translation factors, and current EST sampling has not been adequate enough to identify them. This result may also agree with our recent understanding that the well-studied TATA-dependent transcription initiation only has limited usage in tissue-specific expression, rather than a universal mechanism [36].

We evaluated the expression breadth distribution of HK408 genes. In EST data, the expression breadths clearly peaked at the value of 18 (Figure 2C). Although only 278 (68.1%) of HK408 genes were found expressed in all 18 tissues due to 5 poorly sampled tissues, 379 (92.9%) were detected in at least 16 of 18 tissues (Table 1). This result justified HK408 genes as a qualified benchmark. In contrast, microarray data detected only 182 (45.3%) and 235 (58.5%) of HK408 genes in all 18 tissues and at lest 16 of 18 tissues, respectively (Table 1). A messy tail of expression breadth distribution across all breadth groups indicated the rather noisy nature and high FN rate in microarray data (Figure 2D).

**Figure 3:**
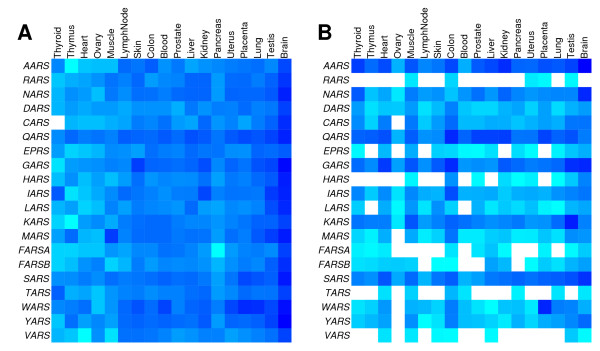
**Expression profiles of 20 human tRNA synthetases in 18 tissues**. Rows and columns of the matrix represent genes and tissues, respectively. Tissues are ranked from the poorly-sampled (left) to the highly-sampled (right) according to the EST data. The darkness of the blue color indicates the original EST counts in EST data (A) and expression intensities in microarray data (B). Blank squares indicate absence of detection. Original EST counts are kept to demonstrate the increasing capability of gene identification from poorly-sampled to highly-sampled tissues.

We studied the detailed expression profiles of all HK408 genes in 18 tissues and took tRNA synthetases as a particular case (Figure 3). Although this group of enzymes was known to be absolutely universal for all cell types, only 8 were ubiquitously detected in microarray data. In contrast, all except one were detected in all 18 tissues in EST data. This single failure in EST data was *CARS *in the most poorly sampled thyroid tissue, and it actually had genuine expression according to microarray data. In general, for the rest of HK408 genes, we observed that the failure of detection in EST data primarily occurred in poorly sampled tissues but microarray data showed lower and irregular detectability. The detailed expression profiles of HK408 genes in EST and microarray data were given in Additional file 3.

### A new catalog of housekeeping genes

As only 70% of HK408 genes can be identified by EST data in all 18 tissues due to several poorly sampled tissues, but 93% of HK408 genes have expression breadths enriched at value 16 to 18 (Figure 2C), we set a cutoff at 16 tissues for a less stringent definition of HK genes. As a result, we obtained 3,140 HK genes as a lower bound (expressed in all 18 tissues and with a FN rate of 31.9%) and 6,909 HK genes as an upper bound (expressed in at least 16 of 18 tissues and with a FN rate of 7.1%) according to the EST data. Similarly, we deduced 1,206 HK genes (with a FN rate of 54.7%) and 2,403 HK genes (with a FN rate of 41.5%) for the low and high numbers according to the microarray data. The detailed descriptions of the 6,909 EST-defined and 2,403 microarray-defined HK genes were given in Additional file 4. We compared 6,909 EST-defined and 2,403 microarray-defined HK genes, and found 4,921 (71.2%) and 415 (17.3%) genes unique to each group, respectively (Additional file 1, Figure S3). In addition, 6,909 EST-defined HK genes covered nearly all HK genes in the lists of Warrington (488/501), Hsiao (403/425) and Eisenberg (502/567). However, when comparing with 2,403 microarray-defined HK genes, we found that 18.6%, 17.4%, and 33.5% genes were unique to the Warrington's, Hsiao's, and Eisenberg's lists, respectively (Additional file 1, Figure S3); the consistency among the microarray-derived results was still very low.

### Tissue-specific expression

We found 1,418 and 885 genes expressed in only one of 18 tissues from microarray and EST data, respectively. The microarray data identified more TS genes than the EST data, but many of which were actually expressed in more than one tissue according to the EST data (Figure 1D). We observed a common trend in both data: brain and testis contributed the most TS genes as compared to other tissues. In EST data, about half of TS genes appeared either brain- or testis-specific. The most important observation we had was that thyroid, the least sampled tissue, had 5,403 of 7,263 detected genes (74.4%) defined as HK genes. This indicated that for poorly sampled tissues our knowledge on their transcriptomes was still limited to the most abundant housekeeping genes, and a true definition of tissue-specific expression required much greater efforts in the future.

### False positive and false negative rates

In our analyses, requiring only one EST for justifying positive expression was a potential source of FP, but the limited sampling depth of present EST data prevented us from using a more stringent threshold. In the least sampled tissue (such as thyroid), 2,607 of 7,263 (35.9%) detected genes were sampled only once. If we required > 1 EST to justify positive expression, these poorly-sampled tissues became non-informative and with very high FN rates. When insisting > 2 ESTs, we had 3 tissues suffered severely for the same reason. More seriously, by doing so, the expression breadths of HK genes peaked at the value 16 rather than 18 — most of HK genes can not be detected in all 18 tissues. When the parameter increased to > 4 ESTs, the peak of HK gene expression breadths disappeared — no clear HK gene group existed (Additional file 1, Table S2).

Although insisting on single EST may introduce FP, there were reasons to suggest that our processed EST data should be a reliable indicator for legitimate expression and the FP involved in our EST-defined results were very low. First, we only took account of ESTs that were reliably aligned onto human genome and clustered into RefSeq loci; most dubious ESTs originated from genomic contaminations and cloning artefacts during cDNA library construction were removed (See methods for details). If we ignore the problems in cloning and RNA isolations (actually faced by both EST and microarray methods), EST sampling is advantageous in that no empirical cutoff on signals is needed to indicate positive expression. When erroneous sequences are discarded and only reliable sequences are used, EST-based methods suffer less from FP than microarray-based ones. Second, according to our newly-established transcriptome-sampling model [37], transcripts with certain expression levels have finite probability to be detected at a given sampling stage. Although other high-throughput experiments such as SAGE do introduce erroneous low-frequency tags, for EST data at such a poor sampling depth even those genes detected at low sampling frequency are most likely to be moderately and even abundantly expressed.

**Figure 4:**
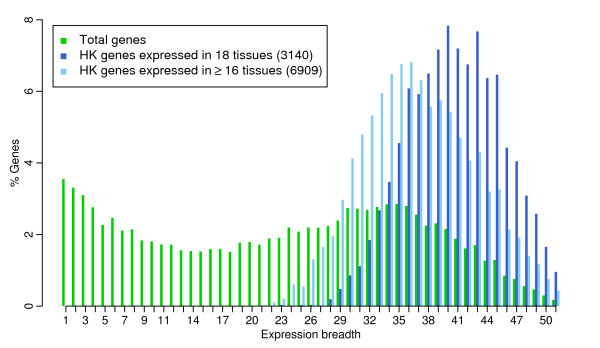
**Validation of EST-defined HK gene in 51 tissues**. Expression breadth distributions in 51 human tissues currently having EST data are compared among total genes and HK genes defined in 18 tissues. The expression breadth distribution of total genes in 51 tissues has two modes representing TS and HK genes respectively, but due to the limited gene detectability in poorly sampled tissues, the spike of HK genes peaks at value 35 and diminishes as tissue broadness increases. The expression breadths of HK genes defined in 18 tissues peak at about value 42 showing very broad expression in 51 tissues.

As among the collection of 3,140 genes each has concrete evidence of expression detected in all 18 tissues, the lower bound of HK genes should be reliable and free of FP. A major source of FP is the expression breadth cutoff value of 16 for defining the upper bound of HK genes. The enrichment of expression breadths at 16 to 18 tissues (Figure 2C) suggested that the FP rate of 6,909 HK genes, if any, should be trivial. Another factor related to FP is that we confined the analyses in only 18 well-studied tissues covered by both microarray and EST data. In order to validate the 3,140 and 6,909 HK genes defined by EST data in 18 tissues, we examined their expression in other tissues presently having EST data. Current EST data covered 51 unique human tissues in total but many of them were very poorly sampled (Additional file 1, Table S1). We observed that the expression breadth distribution in 51 tissues had two modes representing TS and HK genes as what was seen in the 18-tissue collection. However, due to the limited gene detectability in poorly sampled tissues, the expression breadth of HK genes peaked at value 35 and diminished as breadth increased — most of HK genes can only be detected in 35 of 51 tissues (Figure 4). Nevertheless, the expression breadth of HK genes defined in 18 tissues did show very broad expression in 51 tissues — peaked at about value 42 (Figure 4). Therefore, the HK gene list defined in 18 tissues appears very robust and the FP rate should be low.

The high FN rate of microarray data is attributable to the fact that the cutoff value of 200 [19] for defining positive expression is quite conservative. In a parallel analysis, we relaxed the cutoff value to 100 and identified 3,058 and 5,630 genes expressed in all 18 tissues and at least 16 of 18 tissues, respectively, where 66.2% and 78.4% of HK408 genes were covered. These numbers were comparable to those derived from the EST data (3,140 and 6,909). However, as the cutoff value of 200 was determined based on negative controls on the chip to match some optimum ratio of FN to FP, and to our knowledge, almost all published works utilizing this microarray dataset used the cutoff 200, a liberty given to the lower cutoff value for reducing FN should not be encouraged.

## Discussion

At present time, large-scale gene expression profiling is still approached inadequately; both transcriptome sequencing and microarray technique have their own drawbacks. The most noticeable weakness of microarray technique is that it still suffers from poor detectability and reproducibility for low-copy and transiently-expressed genes [38]; the latter are actually very important as they are most likely enzymes and transcription factors, performing transient yet critical biological functions. Systematic noises introduced during sample processing and fluorescence scanning can be improved but are hard to avoid completely, making the cutoff of present/absent call difficult to determine and vary from experiment to experiment. However, EST and its equivalent methods suffer the most from low sampling depth although they are essentially capable of discovering novel and low-copy transcripts [37]. Although about 8 million ESTs have been sequenced, considering the fact that human body has over 200 tissue/cell types, many tissues are still poorly sampled. Fortunately, short tag techniques and recent developments in multiplex sequencing instruments have been applied to comprehensive transcriptome sampling, allowing for effective acquisition of millions of transcript tags in a single experiment [39-41].

Since mRNAs are biological materials of transcriptomes, protocols for RNA isolation and processing are critical for transcriptome studies. Low-copy transcripts may suffer more severely from RNA degradation when lengthy protocols are required, such as making cDNA libraries (especially for SAGE libraries) and labelling RNA probes. These factors make both microarray and EST profile only approximation of *in vivo *expression status. In this *in silicon *study the EST data were generated from 2,563 cDNA libraries, and we consolidated tissue samples from similar origin into uniquely defined tissue. Although precise information of tissue/cell type, which requires advancement of micro-dissection tools and single-cell techniques, has been lost and potential FP may be introduced, this procedure is a necessary approximation considering the limitation of present expression data.

The last but the most profound issue relates to the extended definition of gene itself. Recently, genome-wide tiling array experiments and large-scale full-length cDNA sequencing have provided new insights on the transcribed content of human genome [42,43]. Transcription is complicated by extensive overlap of transcriptional units as well as alternative initiation, splicing and termination; this complex transcriptional organization challenges the traditional definition of a "gene", suggesting that transcripts should be used as operational units of genomes [44]. Consequently, the concept of "housekeeping" or "maintenance" should be defined in a hierarchical way related to cell types, growth stages, cell cycles as well as various physiological conditions, and in terms of specific transcript variant.

Clearly, we are still at an early stage toward precisely defining the basal and cell-specific transcriptomes. However, we believe that along with the improvement of microarray technology and saturated sequencing of transcriptomes, results from microarray and EST data will converge to a consensus. Intensive transcriptome sequencing for the identification of unknown transcript, followed by extensive microarray experiments under various biological contexts, will give us a great opportunity to precisely define cell-specific human transcriptome in the near future.

## Conclusion

The present EST sampling data was far from adequate; many human tissues were still poorly sampled so that our ability to define TS expression was still very limited. Microarray data, due to a likely over-stringent threshold, showed higher FN rate in comparison with EST data, leading to a significant underestimation of HK genes. Based on EST data, we estimated that about 40.0% of the currently annotated human genes were actually universally expressed, nearly a ten-fold increase as compared to the previous estimates based on microarray data solely.

## Methods

### RefSeq loci

We aligned 24,354 human RefSeq transcripts (NCBI, June 18, 2007 update) onto human genomic sequences (UCSC, hg18) using BLAT [45]. Requiring at least 98% base-pair identity and 95% length coverage, we acquired 24,458 gene features on the genome. Features were clustered into loci based on sharing of splicing site for multi-exon features and overlaps of exon for single-exon features. Finally, 18,225 RefSeq loci — 17,009 (93.3%) multi-exon and 1,216 (6.7%) single-exon — were used in further analyses.

### EST and microarray probe annotation

Human EST sequences and their genomic alignments were downloaded from UCSC annotation database (March 11, 2007 update) [46]. We removed 4,609 cDNA libraries with less than 100 ESTs; the number of ESTs from these libraries contributed only 2.0% (156,378) of the total EST collection. The remaining 4,026 libraries contain 7,801,123 (98.0%) ESTs. After post-processing and filtering, 6,039,131 (77.4%) ESTs can be reliably aligned with at least 96% identity and 80% coverage, revealing 3,327,959 spliced and 2,776,470 unspliced features on the genome. EST features were clustered into RefSeq loci according to the following three steps: (1) 3,186,812 (95.8%) spliced features sharing at least one splicing site with a multi-exon RefSeq locus were first clustered into corresponding locus; (2) 1,570,241 unspliced features that exactly locate in an internal exon, extend the 5'-most exon or extend the 3'-most exon of a multi-exon RefSeq locus were then added; (3) 59,173 unspliced features were finally clustered into single-exon RefSeq loci by requiring at least 1-bp overlap. The remaining 1,288,203 EST features, largely unspliced (89.0%), were regarded as unreliable and discarded from further analyses. We retrieved microarray data from Gene Expression Atlas II [19]. The alignment of exemplar/consensus sequences of the probe sets were acquired from UCSC annotation database (April 13, 2006 update), and clustered into RefSeq loci by using similar procedure as for EST clustering. Eventually, 13,986 RefSeq loci were represented on the chip (Affymetrix U133A coupled with GNF1H) [19].

### Tissue classification

cDNA library information was obtained from CGAP (February 27, 2007 update) [47] and UniGene (March 26, 2007 update) [48], followed by integration and manual curations. Information for microarray samples was retrieved from NCBI's GEO database [49]. Since most available tissue samples are anatomically heterogeneous at present time, we *in silicon *consolidated RNA samples from the same tissue and/or partial tissue samples from entire organs into unique tissues to avoid overlapping results. Finally, 4,026 original cDNA libraries and 79 original microarray tissue samples were categorized into 51 and 31 unique tissues respectively. We selected 18 well-studied tissues covered by both two types of data for analyses. Although previous studies reported more number of tissues assayed than our current study, bulky and pooled tissues were used for overlapping gene expression profiling and often resulted in redundant counts. The 18 tissues used in this study have represented a broad spectrum of differentiated tissue/cell types in the human body (Additional file 1, Figure S2).

### Present/absent call

For EST data, we defined a RefSeq locus as expressed in a given tissue when at least one reliably clustered EST was detected from cDNA libraries of that tissue. No empirical cutoff was enforced. This was justified as we have removed most of dubious ESTs — largely unspliced — originated from genomic contaminations and other experimental artefacts. The ESTs clustered in RefSeq loci are well consistent with the annotated gene structure, thus should reliably indicate the genuine expression. For microarray data, we retrieved the expression intensities of each probe set from NCBI's GEO database. Expression intensities from different probe sets of the same RefSeq locus and from different experiments of the same tissue were averaged. We called a RefSeq locus as expressed if its expression intensity exceeded the cutoff value of 200 as recommended by authors who carried out the experiments [9]. We also loosened the cutoff to 100 for comparative analyses.

### Benchmark of HK genes

Pathway information was acquired from Reactome [20] and KEGG [21]. As a benchmark for comparative analyses, we manually curated 408 genes with well-documented housekeeping functions, including general transcription factors [22,23] and major components of capping and polyadenylation machinery [24-26], spliceosome [27-29], nuclear RNA export complex [30-32], translation machinery [33], cytosolic ribosome [34], and ubiquitin-proteasome proteolytic pathway [35] (Table 1 and Additional file 2).

## Abbreviations

HK, housekeeping; TS, tissue-specific; HK408, 408 manually curated genes that are highly proposed to play housekeeping roles; FN, false negative; FP, false positive.

## Authors' contributions

JY supervised the study and helped to draft the manuscript. JZ executed the study and drafted the manuscript. FH collected the data and participated in analyses. SS and JW participated in analyses, and JW was responsible for project management. All authors read and approved the final manuscript.

## Supplementary Material

Additional file 1 Supplementary figures and tables. Additional file 1 contains supplementary figures and tables in this study. Table S1 shows the tissues covered by cDNA libraries. Table S2 shows the number of genes detected by EST data under different thresholds. Figure S1 shows the comparison among previous microarray-defined HK gene lists. Figure S2 shows the illustration of tissues covered by cDNA libraries. Figure S3 shows the comparisons between EST-defined and microarray-defined HK gene lists in this study. Click here for file

Additional file 2 Detailed descriptions of HK408 genes. Additional file 2 provides detailed descriptions of the 408 manually-curated housekeeping genes (HK408). Click here for file

Additional file 3 Expression profiles of HK408 genes. Additional file 3 provides the expression profiles of HK408 genes in 18 tissues among the EST and microarray data. Click here for file

Additional file 4 Detailed descriptions of 6,909 EST-defined and 2,403 microarray-defined HK genes. Additional file 4 provides detailed descriptions of 6,909 EST-defined and 2,403 microarray-defined HK genes categorized in this study. Click here for file
