# Supplementary material for: How many human genes can be defined as housekeeping with current expression data?
Source: BMC Genomics. 2008 Apr 16;9:172. doi: 10.1186/1471-2164-9-172 (PMC2396180; doi:10.1186/1471-2164-9-172)

Table S1 - Tissues covered by cDNA libraries

	Organ/Tissue/Cell	# Lib [a]	# Seq	# Features	# Loci
**1**	**Brain**	**468**	**1258925**	**1071045**	**15309**
**2**	**Testis**	**108**	**386390**	**325983**	**13915**
**3**	**Lung**	**211**	**396833**	**310060**	**13646**
**4**	**Placenta**	**234**	**338222**	**259063**	**12075**
**5**	**Uterus**	**149**	**271135**	**213253**	**12572**
6	ESCell	16	221210	202953	11775
**7**	**Pancreas**	**27**	**241736**	**201488**	**11388**
8	Eye	49	235339	194873	12820
**9**	**Kidney**	**103**	**241947**	**190668**	**12967**
**10**	**Liver**	**61**	**254201**	**183961**	**11042**
**11**	**Prostate**	**153**	**327651**	**178297**	**11892**
**12**	**Blood**	**184**	**220937**	**177216**	**11091**
**13**	**Colon**	**460**	**247157**	**172770**	**11644**
**14**	**Skin**	**26**	**205817**	**144919**	**10756**
15	Breast	434	208733	142898	11375
**16**	**LymphNode**	**18**	**156051**	**119518**	**9643**
**17**	**Muscle**	**53**	**146405**	**111290**	**10830**
18	Stomach	132	118252	93199	9695
**19**	**Ovary**	**80**	**128093**	**90330**	**9706**
**20**	**Heart**	**22**	**101016**	**84653**	**9878**
21	GermCell	9	94995	84617	10963
**22**	**Thymus**	**12**	**83677**	**77522**	**8360**
23	Bone	19	81516	69234	10124
24	Mouth	137	81389	63861	7772
25	Cartilage	17	67150	60028	9574
**26**	**Thyroid**	**194**	**74370**	**49004**	**7263**
27	Trachea	3	50063	48614	6675
28	Vascular	14	54554	48392	7497
29	Spleen	11	57115	47251	7115
30	Cervix	17	52195	42875	7277
31	BoneMarrow	20	60995	38248	6717
32	Pharynx	76	52703	38101	5764
33	SmallIntestine	8	47158	35996	6916
34	Synovium	11	39247	32919	5442
35	PeripheralNervousSystem (PNS)	9	43266	30630	7604
36	Adrenal	16	40337	29448	6344
37	Bladder	31	40522	25991	6293
38	Larynx	128	38506	24957	4182
39	Parathyroid	1	22987	19831	4772
40	Esophagus	7	20483	18747	4754
41	Ear	3	19110	16724	3431
42	Pituitary	9	22829	14484	3964
43	SalivaryGland	6	23847	14177	3679
44	Adipose	9	15858	14017	4296
45	Tonsil	3	20435	13501	2674
46	UmbilicalCord	7	14343	12652	2893
47	Macrophage	3	12853	12019	2262
48	AmnioticFluid	38	11122	6837	1872
49	Rectum	4	6110	5837	2144
50	Epididymis	25	6808	4988	1482
51	Fibroblast	2	4381	4147	2006
52	Pooled	46	485665	390879	16225
53	Other	43	186930	115548	10408
54	Uncharacterized	100	161554	123916	12747
	Total	4026	7801123	6104429	17593

[a] Only 4026 libraries with >=100 ESTs were used.
NOTE: tissues colored in blue were used in this study.

Table S2 - Number of genes detected by EST data under different thresholds

	Microarray	>=1 EST	>=2 ESTs	>=3 ESTs	>=4 ESTs	>=5 ESTs
**HK Mode of EBD** [a]	18	18	18	16	15	-
**Thyroid** [b]	5409	7263	4656	3183	2287	1721
**Thymus**	3546	8360	5902	4452	3442	2787
**Heart**	8518	9878	7648	6006	4730	3819
**Ovary**	2985	9706	7408	5788	4632	3818
**Muscle**	7777	10830	8274	6401	5108	4121
**LymphNode**	3280	9643	8028	6880	6013	5218
**Skin**	7535	10756	9067	7790	6783	5976
**Colon**	6169	11644	9766	8489	7437	6541
**Blood**	2697	11091	9242	7965	6961	6127
**Prostate**	5488	11892	10047	8660	7582	6683
**Liver**	6259	11042	8893	7401	6273	5397
**Kidney**	7024	12967	11361	10013	8853	7852
**Pancreas**	3759	11388	9993	8840	7885	6971
**Uterus**	5777	12572	11058	9833	8832	7926
**Placenta**	6044	12075	10698	9596	8711	7877
**Lung**	4105	13646	12376	11277	10354	9514
**Testis**	8260	13915	12478	11332	10339	9486
**Brain**	8266	15309	14409	13737	13204	12767

[a] The mode for HK gene in expression breath distribution (there are two modes in expression breadth distribution, one for TS gene, another for HK gene).
[b] The number of genes detected in each tissue by microarray data and EST data under different thresholds.

Figure S1 - Comparison among previous HK gene lists

After updating the annotation of these datasets, there were 501, 425, and 567 HK genes in the lists put together by Warrington, Hsiao and Eisenberg, respectively. Although all of them arrived at a rough estimate of approximately 500 human HK genes, the shared HK genes were found significantly low—only 155 genes were found in all three datasets despite the fact that two of them shared 340 genes due to the utilization of an identical technical platform.

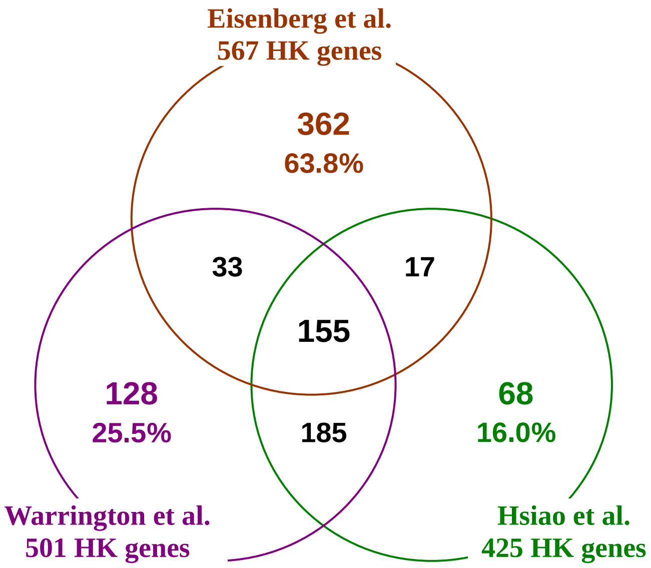

Figure S2 - Illustration of tissues covered by cDNA libraries

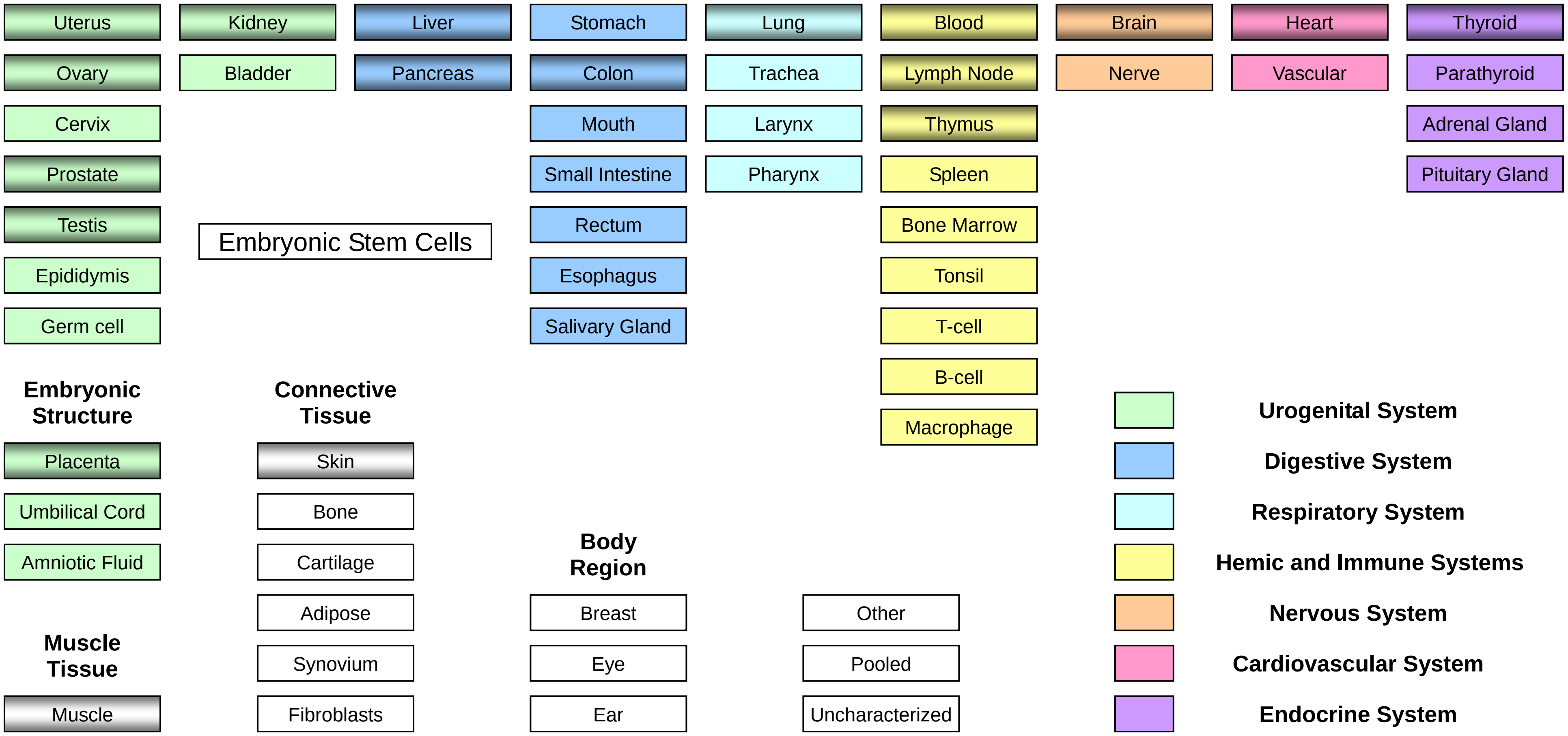

Figure S3 - Comparisons between EST-based and microarray-based HK gene lists

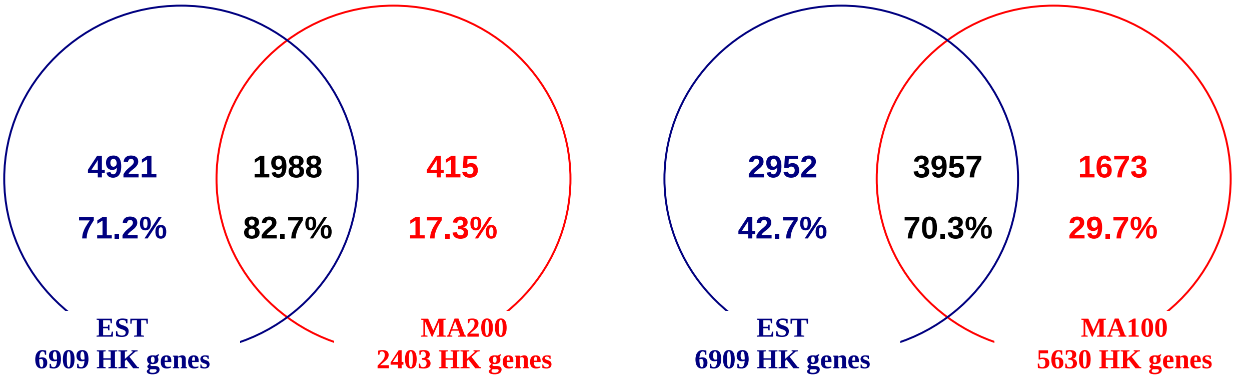

6909 EST HK genes expressed in at least 16 tissues

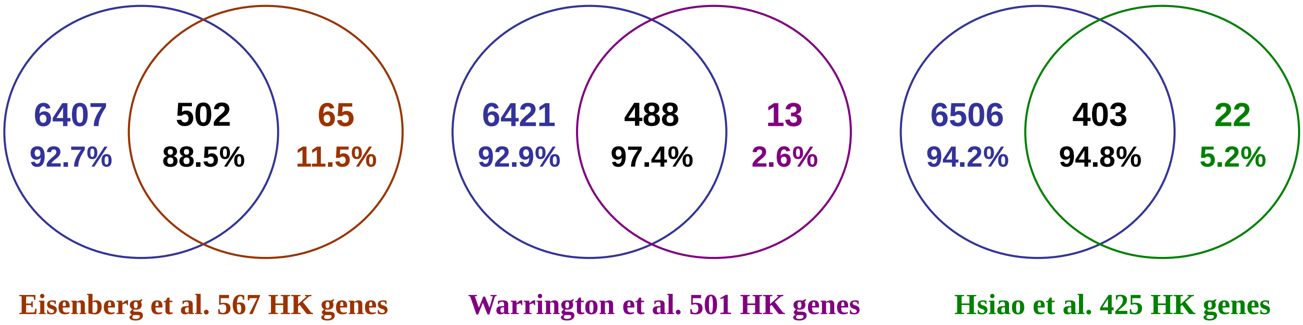

2403 MA200 HK genes expressed in at least 16 tissues

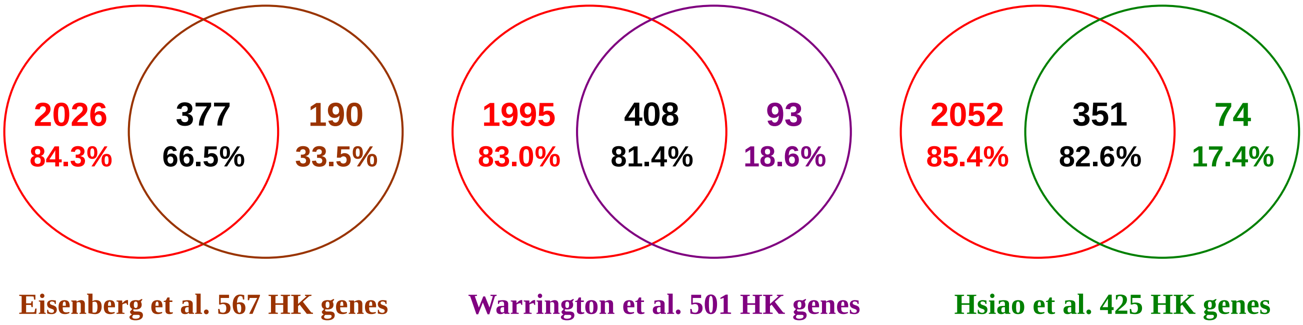

5630 MA100 HK genes expressed in at least 16 tissues

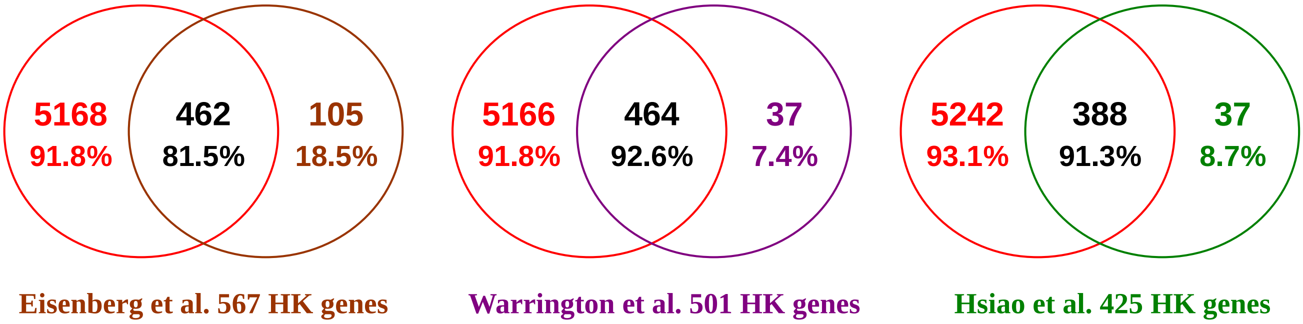

Supplement: Additional file 1 — Supplementary figures and tables. Additional file 1 contains supplementary figures and tables in this study. Table S1 shows the tissues covered by cDNA libraries. Table S2 shows the number of genes detected by EST data under different thresholds. Figure S1 shows the comparison among previous microarray-defined HK gene lists. Figure S2 shows the illustration of tissues covered by cDNA libraries. Figure S3 shows the comparisons between EST-defined and microarray-defined HK gene lists in this study. [file 1471-2164-9-172-S1.pdf]
