# Supplementary material for: How many human genes can be defined as housekeeping with current expression data?
Source: BMC Genomics. 2008 Apr 16;9:172. doi: 10.1186/1471-2164-9-172 (PMC2396180; doi:10.1186/1471-2164-9-172)
Supplement: Additional file 2 — Detailed descriptions of HK408 genes. Additional file 2 provides detailed descriptions of the 408 manually-curated housekeeping genes (HK408). [file 1471-2164-9-172-S2.pdf]

Additional File 2

Additional file 2 provides detailed descriptions of the 408 manually-curated housekeeping genes.

Transcription Pre-initiation Complex (PIC) – 40 genes

General Description	Reactome Description	Symbol	GeneID	RefSeq Acc
TFIID	TBP	*TBP*	6908	NM_003194
	Transcription initiation factor TFIID 250 kDa subunit	*TAF1*	6872	NM_138923
	Transcription initiation factor TFIID 135 kDa subunit	*TAF4*	6874	NM_003185
	Transcription initiation factor TFIID 105 kDa subunit	*TAF4B*	6875	NM_005640
	Transcription initiation factor TFIID 100 kDa subunit	*TAF5*	6877	NM_006951
	Transcription initiation factor TFIID 70 kDa subunit	*TAF6*	6878	NM_005641
	Transcription initiation factor TFIID 55 kDa subunit	*TAF7*	6879	NM_005642
	Transcription initiation factor TFIID 31 kDa subunit	*TAF9*	6880	NM_001015892
	Transcription initiation factor TFIID 30 kDa subunit	*TAF10*	6881	NM_006284
	Transcription initiation factor TFIID 28 kDa subunit	*TAF11*	6882	NM_005643
	Transcription initiation factor TFIID 20/15 kDa subunit	*TAF12*	6883	NM_005644
	Transcription initiation factor TFIID 18 kDa subunit	*TAF13*	6884	NM_005645
TFIIB	TFIIB	*GTF2B*	2959	NM_001514
TFIIA	Transcription Factor IIA alpha chain - p35	*GTF2A1*	2957	NM_015859
	Transcription Factor IIA beta chain - p19	*GTF2A1*	2957	NM_015859
	Transcription initiation factor IIA gamma chain	*GTF2A2*	2958	NM_004492
RNA Pol II	RNA Pol II (unphosphorylated) largest subunit	*POLR2A*	5430	NM_000937
	DNA-directed RNA polymerase II 140 kDa polypeptide	*POLR2B*	5431	NM_000938
	DNA-directed RNA polymerase II 33 kDa polypeptide	*POLR2C*	5432	NM_032940
	DNA-directed RNA polymerase II 16 kDa polypeptide	*POLR2D*	5433	NM_004805
	HsRPABC1	*POLR2E*	5434	NM_002695
	HsRPABC2	*POLR2F*	5435	NM_021974
	DNA-directed RNA polymerase II 19 kDa polypeptide	*POLR2G*	5436	NM_002696
	DNA-directed RNA polymerases I, II, and III 17.1 kDa polypeptide	*POLR2H*	5437	NM_006232
	DNA-directed RNA polymerase II 14.5 kDa polypeptide	*POLR2I*	5438	NM_006233
	DNA-directed RNA polymerase II 13.3 kDa polypeptide	*POLR2J*	5439	NM_006234
	DNA-directed RNA polymerases I, II, and III 7.0 kDa polypeptide	*POLR2K*	5440	NM_005034
	HsRPABC5	*POLR2L*	5441	NM_021128
TFIIF	Transcription initiation factor IIF, alpha subunit	*GTF2F1*	2962	NM_002096
	Transcription initiation factor IIF, beta subunit	*GTF2F2*	2963	NM_004128
TFIIE	Transcription initiation factor IIE, alpha subunit	*GTF2E1*	2960	NM_005513
	Transcription initiation factor IIE, beta subunit	*GTF2E2*	2961	NM_002095
TFIIH	(CAK) Cdk7	*CDK7*	1022	NM_001799
	(CAK) Cyclin H	*CCNH*	902	NM_001239
	(CAK) MAT1	*MNAT1*	4331	NM_002431
	XPD protein	*ERCC2*	2068	NM_000400
	XPB protein	*ERCC3*	2071	NM_000122
	TFIIH basal transcription factor complex p62 subunit	*GTF2H1*	2965	NM_005316
	BTF2-p44 (TFIIH component)	*GTF2H2*	2966	NM_001515
	BTF2-p34 (TFIIH component)	*GTF2H3*	2967	NM_001516
	BTF2-p52 (TFIIH component)	*GTF2H4*	2968	NM_001517

Transcription Elongation Complex – 17 genes

General Description	Reactome Description	Symbol	GeneID	RefSeq Acc
FCP1P	FCP1P protein	*CTDP1*	9150	NM_004715
DSIF	SPT4H1 protein	*SUPT4H1*	6827	NM_003168
	SUPT5H protein	*SUPT5H*	6829	NM_003169
NELF	NELF-A protein	*WHSC2*	7469	NM_005663
	NELF-B protein	*RP13-122B23.3*	25920	NM_015456
	NELF-C/D protein	*TH1L*	51497	NM_198976
	RD protein	*RDBP*	7936	NM_002904
P-TEFb	Cyclin T1	*CCNT1*	904	NM_001240
	Cyclin T2	*CCNT2*	905	NM_058241
	Cdk 9 protein	*CDK9*	1025	NM_001261
TFIIS	TFIIS protein	*TCEA1*	6917	NM_201437
ELL	RNA polymerase II elongation factor ELL	*ELL*	8178	NM_006532
FACT complex	FACT 80 kDa subunit	*SSRP1*	6749	NM_003146
	FACT 140 kDa subunit	*SUPT16H*	11198	NM_007192
Elongin complex	Elongin B protein	*TCEB1*	6921	NM_005648
	Elongin C protein	*TCEB2*	6923	NM_007108
	Elongin A1 protein	*TCEB3*	6924	NM_003198

Essential Splicing Factor – 31 genes

General Description	Reactome Description	Symbol	GeneID	RefSeq Acc
U2AF	U2AF 35 kDa subunit	*U2AF1*	7307	NM_006758
	U2AF 65 kDa subunit	*U2AF2*	11338	NM_007279
Splicing factors	SF2/ASF/SFRS1	*SFRS1*	6426	NM_006924
	SR2 / SC35	*SFRS2*	6427	NM_003016
	SRp20	*SFRS3*	6428	NM_003017
	SR4 / SRp75	*SFRS4*	6429	NM_005626
	SRp40	*SFRS5*	6430	NM_006925
	SRp55	*SFRS6*	6431	NM_006275
	SR7/ 9G8 protein	*SFRS7*	6432	NM_001031684
	SR9 / SRp30	*SFRS9*	8683	NM_003769
	SR 11/ p54	*SFRS11*	9295	NM_004768
	SF1/BBP (Branch binding protein)	*SF1*	7536	NM_201998
	SURP 2, G patch protein	*SF4*	57794	NM_172231
	DDX9/NDH II	*DHX9*	1660	NM_001357
	FUS - RNA binding protein/TLS	*FUS*	2521	NM_004960
	YB-1	*YBX1*	4904	NM_004559
	FLJ10839 DNA-binding protein	*CCAR1*	55749	NM_018237
	RBM 5	*RBM5*	10181	NM_005778
	E1B-AP5 nucleocytoplasmic transport protein	*HNRPUL1*	11100	NM_007040
	CD2BP2 - GYF	*CD2BP2*	10421	NM_006110
	SMC1 (BAA11495)	*SMC1A*	8243	NM_006306
	THOC4(Aly/Ref)	*THOC4*	10189	NM_005782
	SRm160	*SRRM1*	10250	NM_005839
	RNPS1	*RNPS1*	10921	NM_006711
	NFX.1	*NFX1*	4799	NM_002504
	hPrp16	*DHX38*	9785	NM_014003
	hPrp17	*CDC40*	51362	NM_015891
	SPF31 (Dna J)	*DNAJC8*	22826	NM_014280
	hUpf3B	*UPF3B*	65109	NM_023010
Magoh-Y14 complex	magoh	*MAGOH*	4116	NM_002370
	RBM8A/Y14	*RBM8A*	9939	NM_005105

hnRNP – 18 genes

General Description	Reactome Description	Symbol	GeneID	RefSeq Acc
hnRNP	hnRNP A0	*HNRPA0*	10949	NM_006805
	hnRNP A1	*HNRPA1*	3178	NM_031157
	hnRNP A2	*HNRPA2B1*	3181	NM_031243
	hnRNP A3	*HNRPA3*	220988	NM_194247
	hnRNP C1/C2	*HNRPC*	3183	NM_001077442
	hnRNP D0	*HNRPD*	3184	NM_031370
	hnRNP F	*HNRPF*	3185	NM_004966
	hnRNP G	*RBMX*	27316	NM_002139
	hnRNP H	*HNRPH1*	3187	NM_005520
	hnRNP H'	*HNRPH2*	3188	NM_001032393
	hnRNP I	*PTBP1*	5725	NM_031991
	hnRNP K	*HNRPK*	3190	NM_031262
	hnRNP L	*HNRPL*	3191	NM_001533
	hnRNP M	*HNRPM*	4670	NM_005968
	hnRNP R	*HNRPR*	10236	NM_005826
	hnRNP U	*HNRPU*	3192	NM_004501
	hnRNP-E1	*PCBP1*	5093	NM_006196
	hnRNP-E2	*PCBP2*	5094	NM_005016

U1, U2, U4, U5, U6, U11, U12 snRNP – 32 genes

General Description	Reactome Description	Symbol	GeneID	RefSeq Acc
snRNP core proteins	Sm Protein B	*SNRPB*	6628	NM_198216
	Sm protein D1	*SNRPD1*	6632	NM_006938
	Sm protein D2	*SNRPD2*	6633	NM_004597
	Sm protein D3	*SNRPD3*	6634	NM_004175
	Sm protein E	*SNRPE*	6635	NM_003094
	Sm protein F	*SNRPF*	6636	NM_003095
	Sm protein G	*SNRPG*	6637	NM_003096
U1 snRNP specific protein	U1 - 70K protein	*SNRP70*	6625	NM_003089
	U1 - A protein	*SNRPA*	6626	NM_004596
	U1 - C protein	*SNRPC*	6631	NM_003093
U2 snRNP specific protein	U2 A` protein	*SNRPA1*	6627	NM_003090
	U2 B` protein	*SNRPB2*	6629	NM_003092
	SAP 114 (Splicing factor 3A subunit 1)	*SF3A1*	10291	NM_005877
	SAP 60 (splicing factor 3A subunit2)	*SF3A2*	8175	NM_007165
	SAP 61 (Splicing factor 3A subunit 3)	*SF3A3*	10946	NM_006802
	SAP155	*SF3B1*	23451	NM_012433
	SAP 145/ SF3b	*SF3B2*	10992	NM_006842
	SAP130	*SF3B3*	23450	NM_012426
	SAP49	*SF3B4*	10262	NM_005850
	Splicing factor 3B subunit 5	*SF3B5*	83443	NM_031287
	p14 branch site protein	*SF3B14*	51639	NM_016047
	SF3b14b	*PHF5A*	84844	NM_032758
U4:U5:U6 tri-snRNP specific protein	tri-snRNP 15.5 kDa protein	*NHP2L1*	4809	NM_005008
	hPrp4	*PRPF4*	9128	NM_004697
	Sm-like protein LSm2	*LSM2*	57819	NM_021177
	DIM1	*TXNL4A*	10907	NM_006701
	U5-40 kDa protein	*WDR57*	9410	NM_004814
	U5-100 kDa protein	*DDX23*	9416	NM_004818
	U5-102 kDa protein	*PRPF6*	24148	NM_012469
	U5-116 kDa protein	*EFTUD2*	9343	NM_004247
	U5-200kDa helicase	*ASCC3L1*	23020	NM_014014
	hPrp8	*PRPF8*	10594	NM_006445

Capping Related Genes – 5 genes

General Description	Reactome Description	Symbol	GeneID	RefSeq Acc
RPT and GT	mRNA capping enzyme	*RNGTT*	8732	NM_003800
SPT5	Spt5 (suppressor of Ty 5 homolog)	*SUPT5H*	6829	NM_003169
MT	RNA (guanine-7-) methyltransferase	*RNMT*	8731	NM_003799
Cap-binding complex	CBP80	*NCBP1*	4686	NM_002486
	CBP20	*NCBP2*	22916	NM_007362

Cleavage and Polyadenylation Complex – 13 genes

General Description	Reactome Description	Symbol	GeneID	RefSeq Acc
CPSF	CPSF1	*CPSF1*	29894	NM_013291
	CPSF2	*CPSF2*	53981	NM_017437
	CPSF3	*CPSF3*	51692	NM_016207
	CPSF4 - 30 kDa subunit	*CPSF4*	10898	NM_006693
CSTF	CSTF 50 kDa subunit	*CSTF1*	1477	NM_001324
	CSTF 64 kDa subunit	*CSTF2*	1478	NM_001325
	CSTF 77 kD subunit	*CSTF3*	1479	NM_001326
CF I	CF I - 25 kDa subunit	*NUDT21*	11051	NM_007006
	CF I - 59 kDa subunit	*FLJ12529*	79869	NM_024811
CF II	CF II - Clp1	*CLP1*	10978	NM_006831
	CF II - PCF11	*PCF11*	51585	NM_015885
Poly(A) polymerase	Poly(A) polymerase alpha	*PAPOLA*	10914	NM_032632
Poly(A) binding protein	PABPN1	*PABPN1*	8106	NM_004643

Nuclear Pore Complex (NPC) – 29 genes

General Description	Reactome Description	Symbol	GeneID	RefSeq Acc
Nuclear pore complexes (NPC)	Nup107 [nuclear envelope]	*NUP107*	57122	NM_020401
	Nup133 [nuclear envelope]	*NUP133*	55746	NM_018230
	Nup153 [nucleoplasm]	*NUP153*	9972	NM_005124
	Nup155 [nuclear envelope]	*NUP155*	9631	NM_004298
	Nup160 [nuclear envelope]	*NUP160*	23279	NM_015231
	Nup188 [nuclear envelope]	*NUP188*	23511	NM_015354
	Nup205 [nuclear envelope]	*NUP205*	23165	NM_015135
	Gp210 [nuclear envelope]	*NUP210*	23225	NM_024923
	Nup214 [cytosol]	*NUP214*	8021	NM_005085
	Nup35 [nuclear envelope]	*NUP35*	129401	NM_138285
	Nup37 [nuclear envelope]	*NUP37*	79023	NM_024057
	Nup43 [nuclear envelope]	*NUP43*	348995	NM_198887
	Nup50 [nucleoplasm]	*NUP50*	10762	NM_007172
	Nup54 [nuclear envelope]	*NUP54*	53371	NM_017426
	Nup62 [nuclear envelope]	*NUP62*	23636	NM_012346
	Nup75 [nuclear envelope]	*NUP85*	79902	NM_024844
	Nup88 [cytosol]	*NUP88*	4927	NM_002532
	Nup93 [nuclear envelope]	*NUP93*	9688	NM_014669
	Nup96 [nuclear envelope]	*NUP98*	4928	NM_139131
	Nup98 [nuclear envelope]	*NUP98*	4928	NM_005387
	Nup58 [nuclear envelope]	*NUPL1*	9818	NM_014089
	Nup45 [nuclear envelope]	*NUPL1*	9818	NM_014089
	NLP1/CG1 [cytosol]	*NUPL2*	11097	NM_007342
	ALADIN [nuclear envelope]	*AAAS*	8086	NM_015665
	POM121 [nuclear envelope]	*POM121*	9883	NM_172020
	Rae1/Gle2 [nuclear envelope]	*RAE1*	8480	NM_003610
	Nup358 [cytosol]	*RANBP2*	5903	NM_006267
	Seh1 [nuclear envelope]	*SEH1L*	81929	NM_031216
	Tpr [nucleoplasm]	*TPR*	7175	NM_003292

Translation Initiation, Elongation, and Termination Factor – 37 genes

General Description	Reactome Description	Symbol	GeneID	RefSeq Acc
eIF1	eIF1	*EIF1*	10209	NM_005801
eIF1A	eIF1A	*EIF1AX*	1964	NM_001412
eIF2 subunit complex	eIF2-alpha	*EIF2S1*	1965	NM_004094
	eIF2-alpha	*EIF2S2*	8894	NM_003908
	eIF2-gamma	*EIF2S3*	1968	NM_001415
eIF2B subunits complex	eIF2B-alpha	*EIF2B1*	1967	NM_001414
	eIF2B-beta	*EIF2B2*	8892	NM_014239
	eIF2B-gamma	*EIF2B3*	8891	NM_020365
	eIF2B-delta	*EIF2B4*	8890	NM_015636
	eIF2B-epsilon	*EIF2B5*	8893	NM_003907
eIF3 subunit complex	eIF3-alpha	*EIF3S1*	8669	NM_003758
	eIF3-beta	*EIF3S2*	8668	NM_003757
	eIF3-gamma	*EIF3S3*	8667	NM_003756
	eIF3-delta	*EIF3S4*	8666	NM_003755
	eIF3-epsilon	*EIF3S5*	8665	NM_003754
	eIF3e	*EIF3S6*	3646	NM_001568
	eIF3-zeta	*EIF3S7*	8664	NM_003753
	eIF3c	*EIF3S8*	8663	NM_003752
	eIF3-eta	*EIF3S9*	8662	NM_003751
	eIF3-theta	*EIF3S10*	8661	NM_003750
	eIF3k	*EIF3S12*	27335	NM_013234
eIF4F	eIF4A-I	*EIF4A1*	1973	NM_001416
	eIF4A-II	*EIF4A2*	1974	NM_001967
	eIF4E	*EIF4E*	1977	NM_001968
	4E-BP	*EIF4EBP1*	1978	NM_004095
	eIF4G	*EIF4G1*	1981	NM_004953
	eIF4B	*EIF4B*	1975	NM_001417
	eIF4H	*EIF4H*	7458	NM_022170
	eIF5	*EIF5*	1983	NM_001969
	eIF5B	*EIF5B*	9669	NM_015904
eEF1A	eEF1A	*EEF1A1*	1915	NM_001402
eEF2	eEF2	*EEF2*	1938	NM_001961
eEF1B subunit complex	eEF1B alpha	*EEF1B2*	1933	NM_001959
	eEF1B beta	*EEF1D*	1936	NM_032378
	eEF1B gamma	*EEF1G*	1937	NM_001404
eRF1	Eukaryotic peptide chain release factor subunit 1	*ETF1*	2107	NM_004730
eRF3	eRF3	*GSPT2*	23708	NM_018094

tRNA Synthetase – 20 genes

General Description	Description	Symbol	GeneID	RefSeq Acc
	alanyl-tRNA synthetase	AARS	16	NM_001605
	cysteinyl-tRNA synthetase	CARS	833	NM_139273
	aspartyl-tRNA synthetase	DARS	1615	NM_001349
	glutamyl-prolyl-tRNA synthetase	EPRS	2058	NM_004446
	phenylalanyl-tRNA synthetase, alpha subunit	FARSA	2193	NM_004461
	phenylalanyl-tRNA synthetase, beta subunit	FARSB	10056	NM_005687
	glycyl-tRNA synthetase	GARS	2617	NM_002047
	histidyl-tRNA synthetase	HARS	3035	NM_002109
	isoleucyl-tRNA synthetase	IARS	3376	NM_002161
	lysyl-tRNA synthetase	KARS	3735	NM_005548
	leucyl-tRNA synthetase	LARS	51520	NM_020117
	methionyl-tRNA synthetase	MARS	4141	NM_004990
	asparaginyl-tRNA synthetase	NARS	4677	NM_004539
	glutaminyl-tRNA synthetase	QARS	5859	NM_005051
	arginyl-tRNA synthetase	RARS	5917	NM_002887
	seryl-tRNA synthetase	SARS	6301	NM_006513
	threonyl-tRNA synthetase	TARS	6897	NM_152295
	valyl-tRNA synthetase	VARS	7407	NM_006295
	tryptophanyl-tRNA synthetase	WARS	7453	NM_004184
	tyrosyl-tRNA synthetase	YARS	8565	NM_003680

Cytosolic Ribosome – 82 genes

General Description	Reactome Description	Symbol	GeneID	RefSeq Acc
40S ribosomal complex	40S small ribosomal protein 2	RPS2	6187	NM_002952
	40S small ribosomal protein 3	RPS3	6188	NM_001005
	40S small ribosomal protein 3A	RPS3A	6189	NM_001006
	40S small ribosomal protein 4	RPS4X	6191	NM_001007
	40S small ribosomal protein 4Y	RPS4Y1	6192	NM_001008
	40S small ribosomal protein 5	RPS5	6193	NM_001009
	40S small ribosomal protein 6	RPS6	6194	NM_001010
	40S small ribosomal protein 7	RPS7	6201	NM_001011
	40S small ribosomal protein 8	RPS8	6202	NM_001012
	40S small ribosomal protein 9	RPS9	6203	NM_001013
	40S small ribosomal protein 10	RPS10	6204	NM_001014
	40S small ribosomal protein 11	RPS11	6205	NM_001015
	40S small ribosomal protein 12	RPS12	6206	NM_001016
	40S small ribosomal protein 13	RPS13	6207	NM_001017
	40S small ribosomal protein 14	RPS14	6208	NM_005617
	40S small ribosomal protein 15	RPS15	6209	NM_001018
	40S small ribosomal protein 1A	RPS15A	6210	NM_001019
	40S small ribosomal protein 16	RPS16	6217	NM_001020
	40S small ribosomal protein 17	RPS17	6218	NM_001021
	40S small ribosomal protein 18	RPS18	6222	NM_022551
	40S small ribosomal protein 19	RPS19	6223	NM_001022
	40S small ribosomal protein 20	RPS20	6224	NM_001023
	40S small ribosomal protein 21	RPS21	6227	NM_001024
	40S small ribosomal protein 23	RPS23	6228	NM_001025
	40S small ribosomal protein 24	RPS24	6229	NM_001026
	40S small ribosomal protein 25	RPS25	6230	NM_001028
	40S small ribosomal protein 26	RPS26	6231	NM_001029
	40S small ribosomal protein 27	RPS27	6232	NM_001030
	40S small ribosomal protein 27A	RPS27A	6233	NM_002954
	40S small ribosomal protein 28	RPS28	6234	NM_001031
	40S small ribosomal protein 29	RPS29	6235	NM_001032
	40S small ribosomal protein 30	FAU	2197	NM_001997
	40S small ribosomal protein P4	RPSA	3921	NM_002295
60S ribosomal complex	60S acidic ribosomal protein P0	RPLP0	6175	NM_001002
	60S acidic ribosomal protein P1	RPLP1	6176	NM_001003
	60S acidic ribosomal protein P2	RPLP2	6181	NM_001004
	60S ribosomal protein L3	RPL3	6122	NM_000967
	60S ribosomal protein L3-like	RPL3L	6123	NM_005061
	60S ribosomal protein L4	RPL4	6124	NM_000968
	60S ribosomal protein L5	RPL5	6125	NM_000969
	60S ribosomal protein L6	RPL6	6128	NM_000970
	60S ribosomal protein L7	RPL7	6129	NM_000971
	60S ribosomal protein L7a	RPL7A	6130	NM_000972
	60S ribosomal protein L8	RPL8	6132	NM_000973
	60S ribosomal protein L9	RPL9	6133	NM_000661
	60S ribosomal protein L10	RPL10	6134	NM_006013
	60S ribosomal protein L10a	RPL10A	4736	NM_007104
	60S ribosomal protein L11	RPL11	6135	NM_000975
	60S ribosomal protein L12	RPL12	6136	NM_000976
	60S ribosomal protein L13	RPL13	6137	NM_000977
	60S ribosomal protein L13a	RPL13A	23521	NM_012423
	60S ribosomal protein L14	RPL14	9045	NM_003973
	60S ribosomal protein L15	RPL15	6138	NM_002948
	60S ribosomal protein L17	RPL17	6139	NM_000985
	60S ribosomal protein L18	RPL18	6141	NM_000979
	60S ribosomal protein L18a	RPL18A	6142	NM_000980
	60S ribosomal protein L19	RPL19	6143	NM_000981
	60S ribosomal protein L21	RPL21	6144	NM_000982
	60S ribosomal protein L22	RPL22	6146	NM_000983
	60S ribosomal protein L23	RPL23	9349	NM_000978
	60S ribosomal protein L23a	RPL23A	6147	NM_000984
	60S ribosomal protein L24	RPL24	6152	NM_000986
	60S ribosomal protein L26	RPL26	6154	NM_000987
	60S ribosomal protein L26-like 1	RPL26L1	51121	NM_016093
	60S ribosomal protein L27	RPL27	6155	NM_000988
	60S ribosomal protein L27a	RPL27A	6157	NM_000990
	60S ribosomal protein L28	RPL28	6158	NM_000991
	60S ribosomal protein L29	RPL29	6159	NM_000992
	60S ribosomal protein L30	RPL30	6156	NM_000989
	60S ribosomal protein L31	RPL31	6160	NM_000993
	60S ribosomal protein L32	RPL32	6161	NM_000994
	60S ribosomal protein L34	RPL34	6164	NM_000995
	60S ribosomal protein L35	RPL35	11224	NM_007209
	60S ribosomal protein L35a	RPL35A	6165	NM_000996
	60S ribosomal protein L36	RPL36	25873	NM_015414
	60S ribosomal protein L44	RPL36A	6173	NM_021029
	60S ribosomal protein L37	RPL37	6167	NM_000997
	60S ribosomal protein L37a	RPL37A	6168	NM_000998
	60S ribosomal protein L38	RPL38	6169	NM_000999
	60S ribosomal protein L39	RPL39	6170	NM_001000
	60S ribosomal protein L41	RPL41	6171	NM_001035267
	60S ribosomal protein L40	UBA52	7311	NM_003333

Ubiquitin Mediated Proteolysis – 45 genes

General Description	Description	Symbol	GeneID	RefSeq Acc
	cell division cycle 20 homolog (S. cerevisiae)	CDC20	991	NM_001255
	cell division cycle 27 homolog (S. cerevisiae)	CDC27	996	NM_001256
	cell division cycle 34 homolog (S. cerevisiae)	CDC34	997	NM_004359
	neural precursor cell expressed, developmentally down-regulated 4	NEDD4	4734	NM_006154
	S-phase kinase-associated protein 1A (p19A)	SKP1A	6500	NM_006930
	S-phase kinase-associated protein 2 (p45)	SKP2	6502	NM_005983
	transcription elongation factor B (SIII), polypeptide 1 (15kDa, elongin C)	TCEB1	6921	NM_005648
	transcription elongation factor B (SIII), polypeptide 2 (18kDa, elongin B)	TCEB2	6923	NM_007108
	ubiquitin-activating enzyme E1 (A1S9T and BN75 temperature sensitivity complementing)	UBE1	7317	NM_003334
	ubiquitin-conjugating enzyme E2D 1 (UBC4/5 homolog, yeast)	UBE2D1	7321	NM_003338
	ubiquitin-conjugating enzyme E2D 2 (UBC4/5 homolog, yeast)	UBE2D2	7322	NM_003339
	ubiquitin-conjugating enzyme E2D 3 (UBC4/5 homolog, yeast)	UBE2D3	7323	NM_003340
	ubiquitin-conjugating enzyme E2E 1 (UBC4/5 homolog, yeast)	UBE2E1	7324	NM_003341
	ubiquitin-conjugating enzyme E2E 2 (UBC4/5 homolog, yeast)	UBE2E2	7325	NM_152653
	von Hippel-Lindau tumor suppressor	VHL	7428	NM_000551
	cullin 3	CUL3	8452	NM_003590
	cullin 2	CUL2	8453	NM_003591
	cullin 1	CUL1	8454	NM_003592
	cell division cycle 23 homolog (S. cerevisiae)	CDC23	8697	NM_004661
	cell division cycle 16 homolog (S. cerevisiae)	CDC16	8881	NM_003903
	hect domain and RLD 2	HERC2	8924	NM_004667
	hect (homologous to the E6-AP (UBE3A) carboxyl terminus) domain and RCC1 (CHC1)-like domain (RLD) 1	HERC1	8925	NM_003922
	beta-transducin repeat containing	BTRC	8945	NM_003939
	ring-box 1	RBX1	9978	NM_014248
	anaphase promoting complex subunit 10	ANAPC10	10393	NM_014885
	ubiquitin-conjugating enzyme E2E 3 (UBC4/5 homolog, yeast)	UBE2E3	10477	NM_006357
	WW domain containing E3 ubiquitin protein ligase 1	WWP1	11059	NM_007013
	WW domain containing E3 ubiquitin protein ligase 2	WWP2	11060	NM_007014
	ubiquitin-conjugating enzyme E2C	UBE2C	11065	NM_007019
	F-box and WD repeat domain containing 11	FBXW11	23291	NM_012300
	neural precursor cell expressed, developmentally down-regulated 4-like	NEDD4L	23327	NM_015277
	anaphase promoting complex subunit 2	ANAPC2	29882	NM_013366
	anaphase promoting complex subunit 4	ANAPC4	29945	NM_013367
	fizzy/cell division cycle 20 related 1 (Drosophila)	FZR1	51343	NM_016263
	ubiquitin protein ligase E3 component n-recognin 5	UBR5	51366	NM_015902
	anaphase promoting complex subunit 5	ANAPC5	51433	NM_016237
	anaphase promoting complex subunit 7	ANAPC7	51434	NM_016238
	APC11 anaphase promoting complex subunit 11 homolog (yeast)	ANAPC11	51529	NM_016476
	ubiquitin-conjugating enzyme E2D 4 (putative)	UBE2D4	51619	NM_015983
	ubiquitin-conjugating enzyme E2R 2	UBE2R2	54926	NM_017811
	F-box and WD repeat domain containing 7	FBXW7	55294	NM_033632
	SMAD specific E3 ubiquitin protein ligase 1	SMURF1	57154	NM_020429
	anaphase promoting complex subunit 1	ANAPC1	64682	NM_022662
	SMAD specific E3 ubiquitin protein ligase 2	SMURF2	64750	NM_022739
	itchy homolog E3 ubiquitin protein ligase (mouse)	ITCH	83737	NM_031483

Proteasome – 43 genes

General Description	Description	Symbol	GeneID	RefSeq Acc
	proteasome subunit, alpha type, 1	PSMA1	5682	NM_002786
	proteasome subunit, alpha type, 2	PSMA2	5683	NM_002787
	proteasome subunit, alpha type, 3	PSMA3	5684	NM_002788
	proteasome subunit, alpha type, 4	PSMA4	5685	NM_002789
	proteasome subunit, alpha type, 5	PSMA5	5686	NM_002790
	proteasome subunit, alpha type, 6	PSMA6	5687	NM_002791
	proteasome subunit, alpha type, 7	PSMA7	5688	NM_002792
	proteasome subunit, alpha type, 8	PSMA8	143471	NM_001025096
	proteasome subunit, beta type, 1	PSMB1	5689	NM_002793
	proteasome subunit, beta type, 2	PSMB2	5690	NM_002794
	proteasome subunit, beta type, 3	PSMB3	5691	NM_002795
	proteasome subunit, beta type, 4	PSMB4	5692	NM_002796
	proteasome subunit, beta type, 5	PSMB5	5693	NM_002797
	proteasome subunit, beta type, 6	PSMB6	5694	NM_002798
	proteasome subunit, beta type, 7	PSMB7	5695	NM_002799
	proteasome subunit, beta type, 8	PSMB8	5696	NM_004159
	proteasome subunit, beta type, 9	PSMB9	5698	NM_002800
	proteasome subunit, beta type, 10	PSMB10	5699	NM_002801
	proteasome 26S subunit, ATPase, 1	PSMC1	5700	NM_002802
	proteasome 26S subunit, ATPase, 2	PSMC2	5701	NM_002803
	proteasome 26S subunit, ATPase, 3	PSMC3	5702	NM_002804
	proteasome 26S subunit, ATPase, 4	PSMC4	5704	NM_006503
	proteasome 26S subunit, ATPase, 5	PSMC5	5705	NM_002805
	proteasome 26S subunit, ATPase, 6	PSMC6	5706	NM_002806
	proteasome 26S subunit, non-ATPase, 1	PSMD1	5707	NM_002807
	proteasome 26S subunit, non-ATPase, 2	PSMD2	5708	NM_002808
	proteasome 26S subunit, non-ATPase, 3	PSMD3	5709	NM_002809
	proteasome 26S subunit, non-ATPase, 4	PSMD4	5710	NM_002810
	proteasome 26S subunit, non-ATPase, 5	PSMD5	5711	NM_005047
	proteasome 26S subunit, non-ATPase, 6	PSMD6	9861	NM_014814
	proteasome 26S subunit, non-ATPase, 7	PSMD7	5713	NM_002811
	proteasome 26S subunit, non-ATPase, 8	PSMD8	5714	NM_002812
	proteasome 26S subunit, non-ATPase, 9	PSMD9	5715	NM_002813
	proteasome 26S subunit, non-ATPase, 10	PSMD10	5716	NM_002814
	proteasome 26S subunit, non-ATPase, 11	PSMD11	5717	NM_002815
	proteasome 26S subunit, non-ATPase, 12	PSMD12	5718	NM_002816
	proteasome 26S subunit, non-ATPase, 13	PSMD13	5719	NM_002817
	proteasome 26S subunit, non-ATPase, 14	PSMD14	10213	NM_005805
	proteasome activator subunit 1	PSME1	5720	NM_006263
	proteasome activator subunit 2	PSME2	5721	NM_002818
	proteasome activator subunit 3	PSME3	10197	NM_005789
	proteasome activator subunit 4	PSME4	23198	NM_014614
	proteasome inhibitor subunit 1	PSMF1	9491	NM_006814
