# Supplementary figures and images for: How many human genes can be defined as housekeeping with current expression data?
Source: BMC Genomics. 2008 Apr 16;9:172. doi: 10.1186/1471-2164-9-172 (PMC2396180; doi:10.1186/1471-2164-9-172)

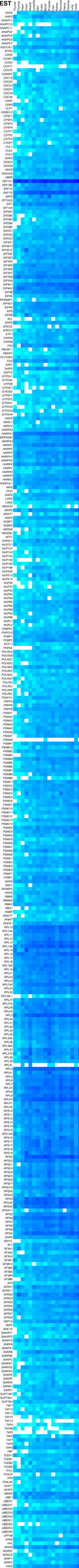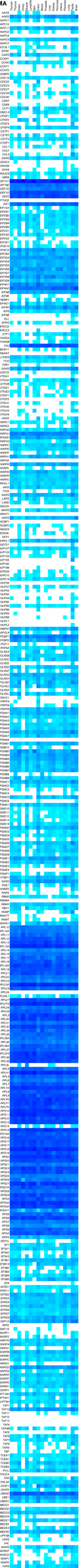

Supplement: Additional file 3 — Expression profiles of HK408 genes. Additional file 3 provides the expression profiles of HK408 genes in 18 tissues among the EST and microarray data. [file 1471-2164-9-172-S3.pdf]
